# Synthesis of indium oxi-sulfide films by atomic layer deposition: The essential role of plasma enhancement

**DOI:** 10.3762/bjnano.4.85

**Published:** 2013-11-13

**Authors:** Cathy Bugot, Nathanaëlle Schneider, Daniel Lincot, Frédérique Donsanti

**Affiliations:** 1 Institut de Recherche et Développement sur l’Energie Photovoltaïque (IRDEP), UMR 7174 – EDF – CNRS – Chimie Paristech, 6 quai Watier, 78401 Chatou, France

**Keywords:** atomic layer deposition, buffer layer, indium oxi-sulfide, plasma enhancement, thin film solar cells

## Abstract

This paper describes the atomic layer deposition of In_2_(S,O)_3_ films by using In(acac)_3_ (acac = acetylacetonate), H_2_S and either H_2_O or O_2_ plasma as oxygen sources. First, the growth of pure In_2_S_3_ films was studied in order to better understand the influence of the oxygen pulses. X-Ray diffraction measurements, optical analysis and energy dispersive X-ray spectroscopy were performed to characterize the samples. When H_2_O was used as the oxygen source, the films have structural and optical properties, and the atomic composition of pure In_2_S_3_. No pure In_2_O_3_ films could be grown by using H_2_O or O_2_ plasma. However, In_2_(S,O)_3_ films could be successfully grown by using O_2_ plasma as oxygen source at a deposition temperature of *T* = 160 °C, because of an exchange reaction between S and O atoms. By adjusting the number of In_2_O_3_ growth cycles in relation to the number of In_2_S_3_ growth cycles, the optical band gap of the resulting thin films could be tuned.

## Introduction

Chalcopyrite-type thin film solar cells that are based on a Cu(In,Ga)Se_2_ (CIGS) absorber have reached high efficiencies, up to 20.3% [1] in 2011 and 20.4% [2] on flexible substrates in 2013. The best efficiencies were obtained by using cadmium sulfide (CdS) as buffer layer in solar cells with a glass/Mo/CIGS/CdS/i-ZnO/ZnO:Al stack. The buffer layer is an n-type semiconductor that forms the p–n junction with the p-type CIGS absorber, and also modifies the CIGS surface chemistry, which is usually too sensitive for a direct deposition of the window layers. However, because of the toxicity of cadmium and the low optical band gap of CdS (2.4 eV [3]) that limits the light conversion of CIGS in the UV range of the solar spectrum, alternative materials have been developed. Most Cd-free buffer layers are based on zinc and indium-compounds, with current record efficiencies obtained by chemical bath deposition (CBD, 19.7% and 19.1% for Zn(S,O,OH) [4–5], 15.7% for In(S,O,OH) [6]) or atomic layer deposition (ALD, 18.5% for Zn(O,S) [7], 18.1% for (Zn,Mg)O [8], 16.4% for In_2_S_3_ [9], and 18.2% for (Zn,Sn)O [10]). Recently, our group has synthesized new mixed films of ZnS/In_2_S_3_ by using ALD and applied them as buffer layers in CIGS solar cells [11–12]. ALD is based on sequential self-saturated reactions that allows the conformal and uniform growth of thin films with a high control of their properties [13–15]. It is therefore a suitable technique for the deposition of buffer layers. Platzer-Björkman et al. have used ALD to improve the energy-band alignment between the CIGS and the front electrode by controlling the oxygen concentration in Zn(S,O) buffer layers [4,16]. Oxygen-doping of In_2_S_3_ films is known to increase their optical band gap value [6,17–18]. Indeed, by O-doping of In_2_S_3_ films deposited by thermal evaporation, Barreau et al. could increase the optical band gap value of In_2_S_3_ thin films from 2.1 to 2.9 eV [17]. In the same way, by using the spray pyrolysis technique, Maha et al. have inserted sulfur atoms in In_2_O_3_ thin films and obtained optical band gaps in the range from 3.85 to 3.96 eV [18]. Thus, based on our previous results and those studies, we became interested in adjusting the optical properties of In_2_S_3_ by incorporating oxygen atoms while using the advantages of ALD. Typical ALD processes for the deposition of In_2_S_3_ and In_2_O_3_ are referenced in Table 1. As ALD processes of In_2_O_3_ report relatively small growth rates, we will consider the case of plasma enhancement. Indeed, plasma-enhanced ALD (PEALD), in which various reactive species are generated, has been the key for the development of fast thin-film deposition processes at low temperature. It is widely used to enhance the thin-film deposition of materials such as Al_2_O_3_, ZnO, Ta_2_O_5_, TiN, TaN and SiN*_x_* [19].

**Table 1:** Typical ALD processes for the deposition of In_2_O_3_ and In_2_S_3_.

reactant A	reactant B	temperature (°C)	growth rate (Å/cycle)	reference

**indium oxide**				

InCl_3_	H_2_O	500	0.27	[20]
InCp	O_3_/O_2_/H_2_O	250	1.3/0.16/0.068	[21]
InCp	H_2_O & O_2_	100–250	1.0–1.6	[22]
TmIn	H_2_O	217	0.39	[23]
In(acac)_3_	H_2_O/O_3_	165–225	0.2/0.12	[24]

**indium sulfide**				

InCl_3_	H_2_S	300	1.4	[25]
In(acac)_3_	H_2_S	160, 180, 160, 150	0.6, 0.7, 0.44, 0.3	[9,26–28]

In this study, ALD and PEALD have been used to synthesize In_2_(S,O)_3_ thin films and carry out optical band-gap engineering. The structural, optical and growth properties of the films will be studied and the role of the plasma will be discussed.

## Results

### Study of In(acac)_3_, H_2_S and H_2_O system

**Figure 1:**
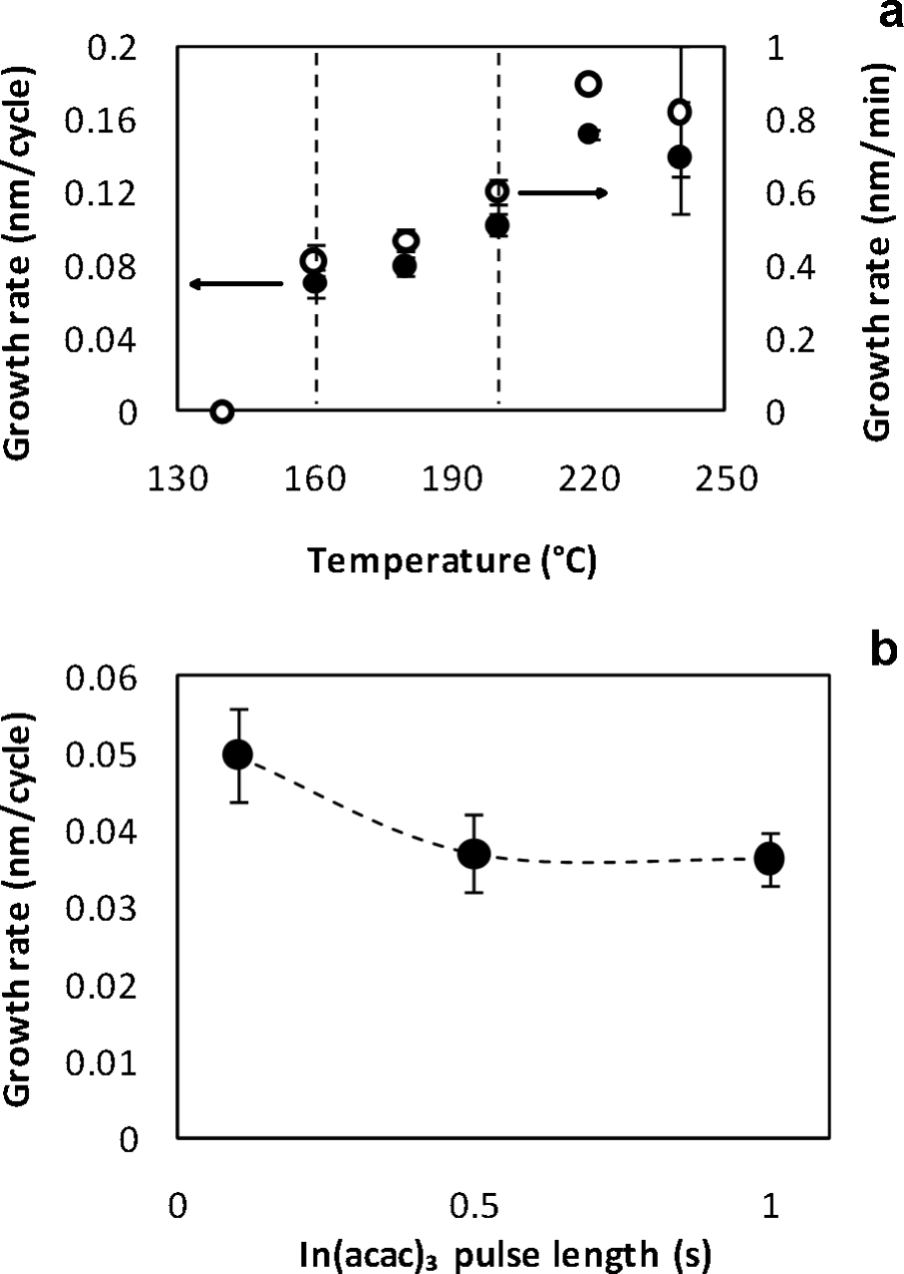
Growth rate of pure In_2_S_3_ a) as function of the process temperature b) as function of the In(acac)_3_ pulse length.

First, a controlled growth of pure In_2_S_3_ films was established and the film properties were measured in order to clearly identify the influence of oxygen pulse later in the study. For that, In_2_S_3_ growth was achieved in the temperature range between 140 and 240 °C. An In_2_S_3_ growth cycle consists of the following steps: In(acac)_3_ exposure/N_2_ purge/H_2_S exposure/N_2_ purge = 0.1/5/0.1/5 s, the relative long purge time being chosen to ensure a good homogeneity. Figure 1a shows the growth rate of In_2_S_3_ thin films at various temperatures. It globally increases with the temperature. An ALD window can be speculatively observed between 160 °C and 200 °C with a mean growth rate of 0.84 Å/cycle. The variation of the In_2_S_3_ growth rate with different In(acac)_3_ pulse lengths at a process temperature of 160 °C is illustrated in Figure 1b. This variation only slightly influences the growth rate and a saturation by lengthening the precursor pulse is not observed. The data suggest that the results displayed on Figure 1a may not have been obtained under completely self-limiting conditions. Structural and optical properties of the films were also investigated. In_2_S_3_ thin films have an amorphous structure for deposition temperatures below 180 °C and a β-tetragonal crystal structure at higher temperatures. Their indirect optical band gap varies from 2.0 eV to 2.2 eV.

**Figure 2:**
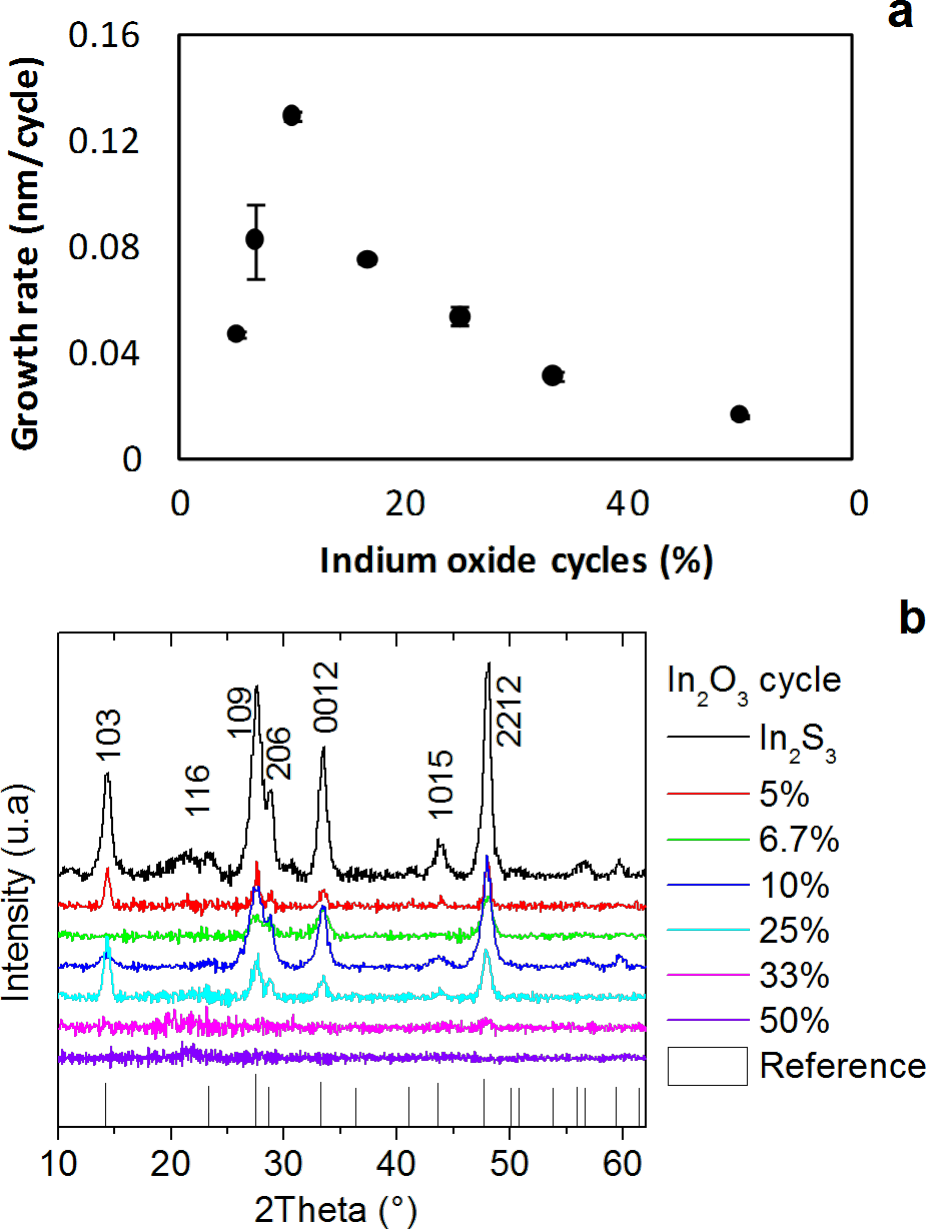
Influence of the number of In_2_O_3_ cycles on (a) the growth rate while using H_2_O as oxygen precursor and (b) GIXRD diffractogram. The reference diffraction pattern for In_2_S_3_ is taken from the database JCPDS 00-005-0731.

Then, we attempted to synthesize In_2_(S,O)_3_ film by inserting an In_2_O_3_ growth cycle. For this H_2_O was pulsed, instead of H_2_S in the growth of pure In_2_S_3_, which led to the supercycles *n·*{In_2_S_3_} + {In_2_O_3_} with *n* = 1,2,3,5,9,14,19, which correspond to ratios of {50%, 33%, 25%, 10%, 6.7%, 5%} of In_2_O_3_ cycles at a deposition temperature of 200 °C. All samples were deposited performing a total of 2000 growth cycles, i.e., 100 supercycles for *n* = 19, 133 supercycles for *n* = 14, etc. Energy dispersive X-ray spectroscopy analysis was performed on the samples and gave atomic ratios of 0.4 for In/(In+S) and 0.6 for S/(In+S), which correspond to typical In_2_S_3_ atomic ratios. The oxygen contents are similar to those of pure In_2_S_3_ films, which is assigned to the oxygen contamination of the substrate. Those results were confirmed by GI-XRD measurements. They were performed to investigate the influence of the H_2_O pulse on the microstructure of the films (Figure 2b). Not all samples were crystalline and the crystalline ones can be attributed to β-In_2_S_3_ with a random orientation by comparing the diffraction patterns with the reference data and with the literature [27]. Indeed, we should observe a peak shift due to increasing oxygen doping when changing the In_2_O_3_/In_2_S_3_ ratio. However, the peaks remain at the same diffraction angles. Comparing the FWHM of the (109) peak, the maximum FWHM measured was 1.2° for the 10%-In_2_O_3_ sample, which corresponds to the thickest film. In general it can be said that the thinner the films, the lower the FWHM. From these observations, it seems that we obtained In_2_S_3_ films only.

**Figure 3:**
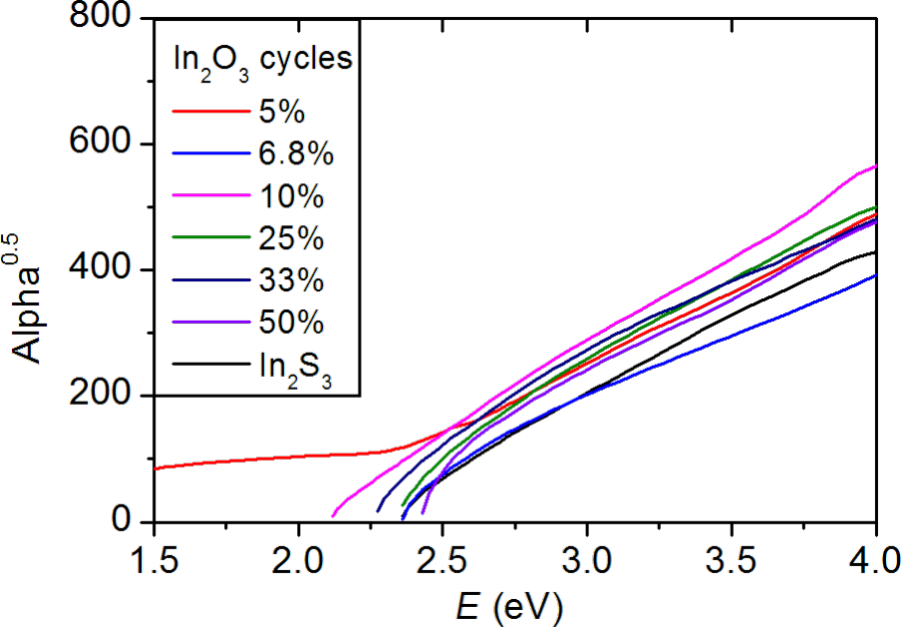
Influence of the ratio of In_2_O_3_ cycles on the film absorption spectra when using H_2_O as oxygen precursor.

Thin films optical absorption were determined from transmittance (*T*) and reflectance (*R*) measurements by using the following formula [29]


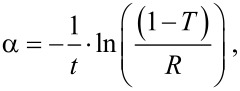


where α is the absorption coefficient and *t* is the film thickness. Figure 3 shows absorption spectra of the thin films. They are presented in the form of (α)^0.5^ = f(*E*), which is linear for indirect band gap materials and allows for the determination of the optical transition. The optical band gaps correspond to an indirect transition in the range from 1.9 to 2.2 eV, which is roughly similar to that of pure In_2_S_3_ film optical properties. No correlation could be found between either the ratio of In_2_O_3_ cycles or the film thickness and the optical measurements. These results are in accordance with the observations of the structural analysis. Consequently, this method is not suitable to synthesize In_2_(S,O)_3_ thin films. In parallel, we attempted to synthesize pure In_2_O_3_ films from In(acac)_3_ and H_2_O at temperatures of 160 and 200 °C. This remained unsuccessful, because no films could be grown under these conditions.

### Study of the In(acac)_3_, H_2_S and O_2_ plasma system

**Figure 4:**
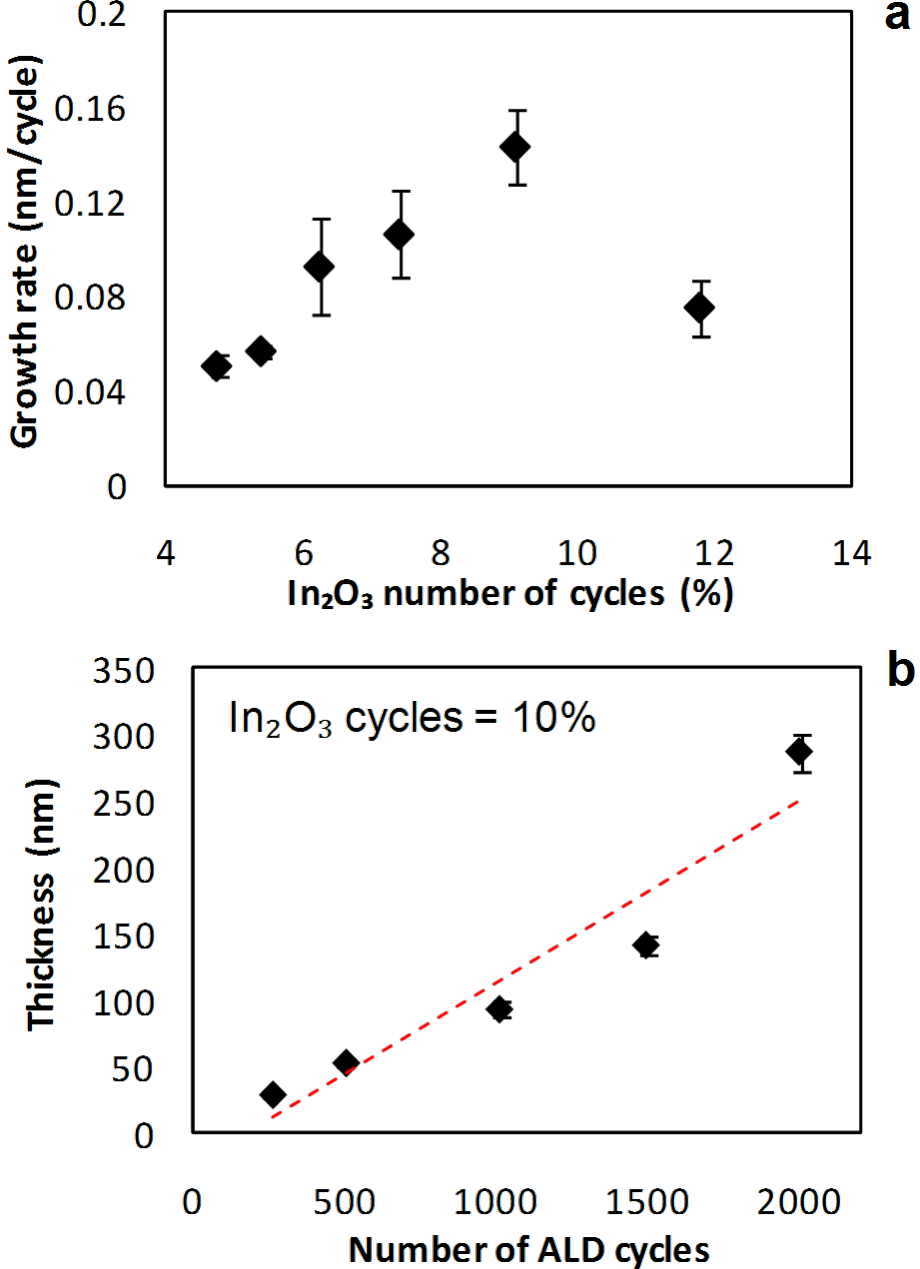
Influence of a) the number of In_2_O_3_ cycles on the growth rate b) the number of process cycles on the film thickness when using O_2_ plasma as oxygen precursor. The dotted line is a guide to the eyes.

As O_2_ plasma is known to have a different reactivity, it was evaluated as potential oxygen source for the deposition of In_2_(S,O)_3_. Thin film syntheses were performed by incorporating In_2_O_3_ growth cycles that use O_2_ plasma pulses in the growth of In_2_S_3_: In(acac)_3_ exposure/N_2_ purge/O_2_ + plasma exposure/N_2_ purge = 0.1/5/7/3 s in the following supercycles *n*·{In_2_S_3_} + 2·{In_2_O_3_} with *n* = 15,20,25,30,35,40 which correspond to ratios of {11.8%, 9.1%, 7.4%, 6.25%, 5.4%, 4.8%} of In_2_O_3_ cycles at a deposition temperature of 160 °C. A total of 2000 cycles was achieved for all samples as described in the previous section. The dependence of the growth rate on the number of In_2_O_3_ cycles is shown in Figure 4a. When increasing the ratio from 4.8% to 9.1%, the growth rate increases up to 1.4 Å/cycle and then decreases again. The variation of the film thickness with the number of ALD cycles for a ratio of 10% of In_2_O_3_ cycles is illustrated in Figure 4b. A linear growth is observed up to 1500 ALD cycles. GIXRD measurements revealed an amorphous structure in all the samples.

**Figure 5:**
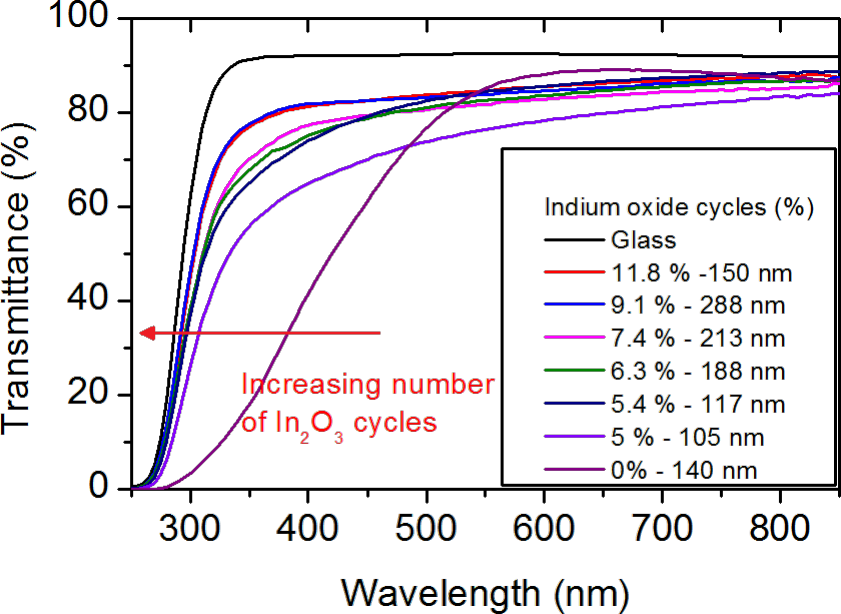
Influence of the number of In_2_O_3_ cycle on the film transmittance when using O_2_ plasma as oxygen precursor.

**Figure 6:**
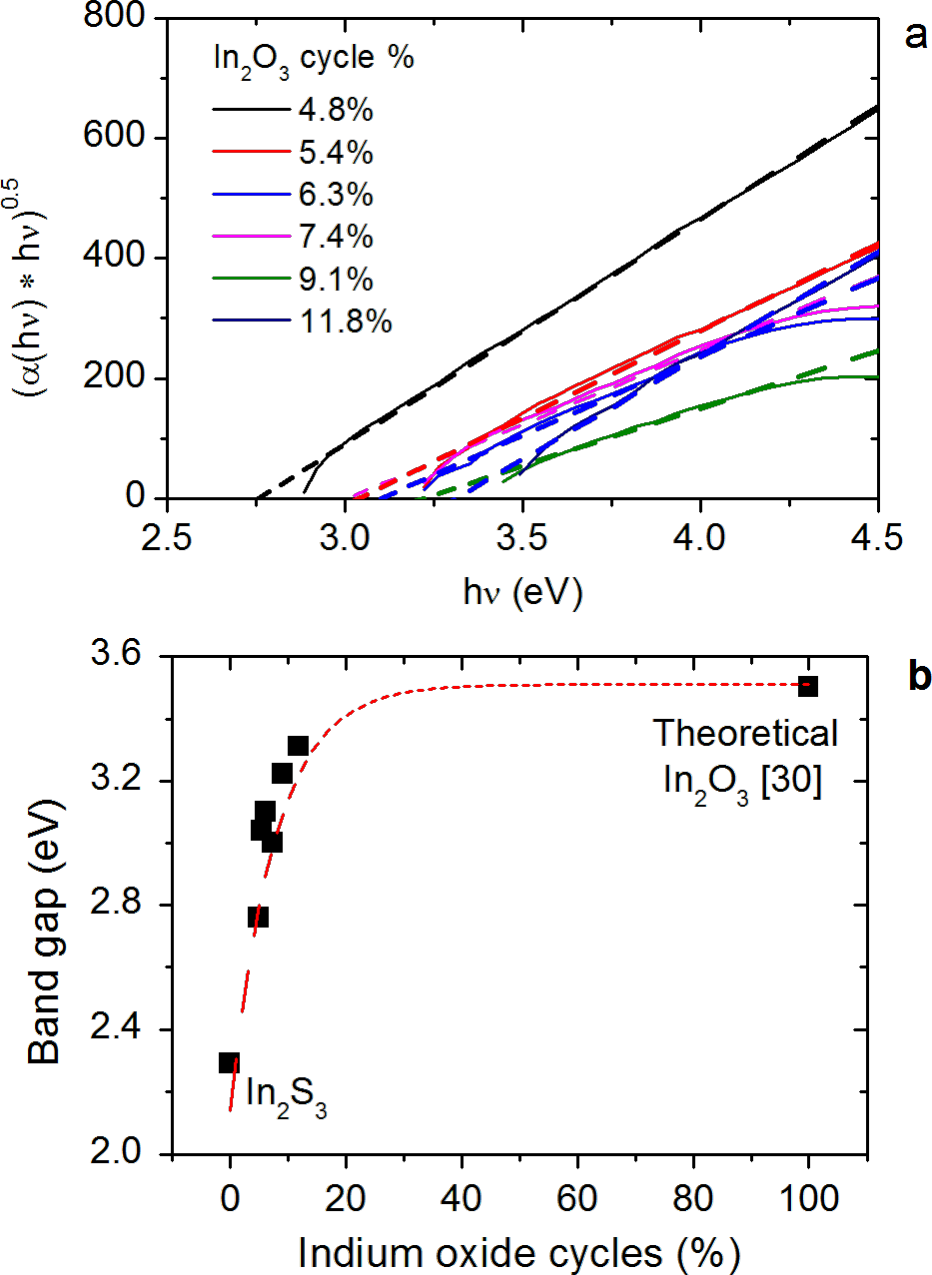
Influence of the number of In_2_O_3_ cycles a) on the absorption and b) optical band gap when using O_2_ plasma as oxygen precursor. The dotted line is a guide to the eyes.

Transmittance and reflectance measurements were carried out on the In_2_(S,O)_3_ samples. Figure 5 shows the transmittance of In_2_(S,O)_3_ films as a function of the percentage of In_2_O_3_ cycles. A shift of the onset absorption can be observed, which suggests an evolution in the properties of the films. The indirect optical transitions were identified for all samples from their respective absorption spectra (Figure 6a). The values are plotted as a function of the ratio of In_2_O_3_ cycles in Figure 6b. The maximum value corresponds to the theoretical optical gap of In_2_O_3_ [30]. The optical band gaps vary from 2.2 ± 0.1 eV for pure In_2_S_3_ to 3.3 ± 0.1 eV for In_2_(S,O)_3_ and increase with the number of In_2_O_3_ cycles during the deposition process of In_2_S_3_.

The atomic ratios of oxygen, sulfur and indium determined by using EDX are presented in Table 2 and correlated to the optical band gap values. The dependence of the atomic ratio of oxygen and the optical band gap on the number of In_2_O_3_ cycles is not clear. In general, high oxygen concentrations of more than 66 atom % were measured in the films.

**Table 2:** EDX measurements data from In_2_(S,O)_3_ thin films when using O_2_ plasma.

In_2_O_3_ (%)	optical band gap (eV)	(O+S)/(In+S+O) (atom %)	In/(In+S+O) (atom %)	S/(In+S+O) (atom %)	O/(In+S+O) (atom %)

4.80	2.76	83	17	15	68
5.41	3.04	85	15	10	75
6.25	3.10	85	15	11	74
7.41	3.00	80	20	14	66
9.09	3.22	79	21	10	69
11.76	3.31	83	17	9	74

We also tried to synthesize In_2_(S,O)_3_ by using a single O_2_ plasma pulse instead of In_2_O_3_ pulse cycles. The following cycle program was used: 20·{In_2_S_3_} + 2·{O_2_+plasma exposure}/N_2_ purge with the same process parameters. This corresponds to 9.1% of indium cycles, which can be compared to the previous deposition with a pulse of In(acac)_3_ before the O_2_ plasma exposure. Table 3 shows the properties of these two samples, along with those of pure In_2_S_3_. Even without the In(acac)_3_ pulse, O and S atomic ratios indicate that the synthesized film corresponds to a In_2_(S,O)_3_ film and no significant differences were observed between the samples.

**Table 3:** Comparison between In_2_(S,O)_3_ films, synthesized with and without In(acac)_3_ during the oxidation pulse, and In_2_S_3_.

program	*E*_g_ (eV)	growth rate (Å/cycle)	In/(In+S+O) (atom %)	S/(S+O) (atom %)	O/(S+O) (atom %)

20·{In_2_S_3_} + 2·{In_2_O_3_}	3.2 ± 0.1	1.4 ± 0.2	21	13	87
20·{In_2_S_3_} + 2·O_2_ plasma	3.3 ± 0.1	1.2 ± 0.2	17	15	85
In_2_S_3_	2.2 ± 0.2	0.7 ± 0.08	35	70	30

The growth of In_2_(S,O)_3_ growth could be achieved when using O_2_ plasma as oxygen precursor. The maximum growth rate was 1.4 Å/cycle, which is higher than the growth rates of In_2_S_3_ shown in Figure 1 and those reported in the literature for this deposition temperature [9,26–28]. Optical measurements revealed an onset absorption moving to higher energies when increasing the number of In_2_O_3_ cycles. At the same time, the optical band gap increased from 2.2 eV to 3.3 eV for In_2_O_3_ cycle ratios in the range from 0 to 11.8%. EDX analysis showed that those films have a high oxygen content. Finally, all attempts to synthesize pure In_2_O_3_ films from In(acac)_3_ and O_2_ plasma remained unsuccessful .

## Discussion

It has been observed that inserting an In_2_O_3_ cycle during the deposition of In_2_S_3_ when using H_2_O as oxygen precursor has no influence on the oxygen content and on the film properties. It only affects the growth rate as the thickness varies. Attempts to synthesize pure In_2_O_3_ thin films were also unsuccessful, which suggests a low reactivity of H_2_O towards In(acac)_3_. Several authors reported the difficulty to synthesize In_2_O_3_ by ALD using β-diketonates (In(acac)_3_, In(hfac = hexafluoropentadionate)_3_, In(thd = 2,2,6,6-tetramethyl-3,5-heptanedioneate)) and water [20–21,25]. In most cases, they assigned the low growth rates or the absence of grown films to the low reactivity of water toward β-diketonates.

As no pure In_2_O_3_ films could be grown in our case, the synthesis of mixed films by a simple addition of two layers, i.e., In_2_O_3_ + In_2_S_3_, is not possible. However, the deposition of ternary materials can also occur via exchange reactions. For instance, when synthesizing zinc indium sulfide (ZIS) thin films, substitution mechanisms between diethylzinc (DEZ) and In_2_S_3_ could be demonstrated [11]. Similar mechanisms also occur when inserting H_2_O in pure ZnS during the growth of Zn(S,O) by using ALD [4,16]. Such processes do not seem to occur in our case, because the In_2_(S,O)_3_ deposition method that uses H_2_O remained unsuccessful. A possible thermodynamic explanation for the unfavorable deposition of In_2_(S,O)_3_ using H_2_O as oxygen precursor is that the following exchange reaction is endothermic and thus unlikely to occur [31].

[1]
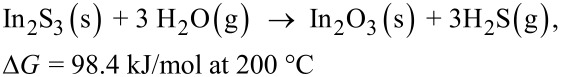


**Figure 7:**
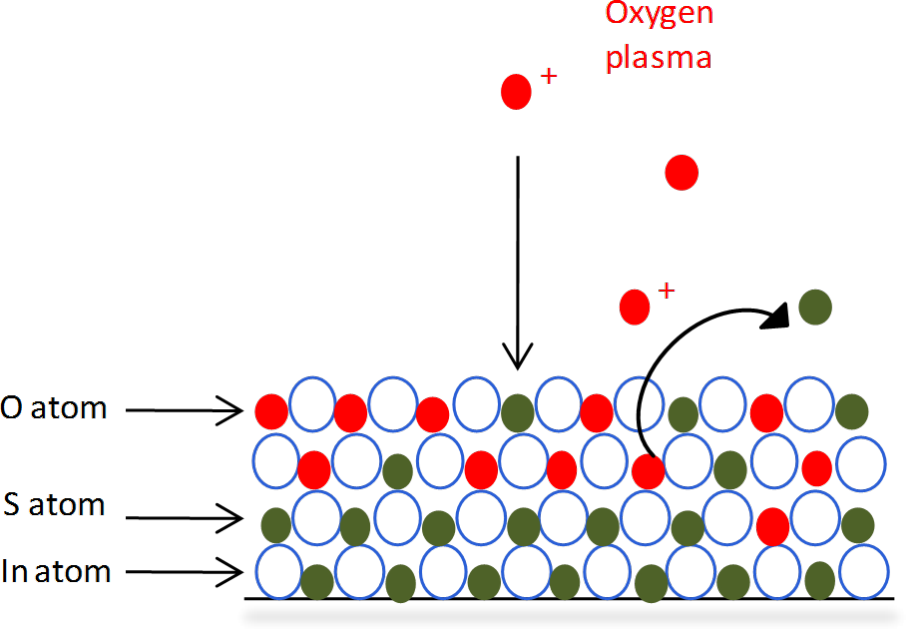
Surface mechanisms during the O_2_ plasma pulse.

Due to the high reactivity of radicals, PEALD generally allows the achievement of many chemical reactions that cannot occur with thermal ALD [13,19]. Here In_2_(S,O)_3_ films could be grown while using O_2_ plasma as oxygen source. But the growth of pure In_2_O_3_ films remained unsuccessful. This suggests that the oxygen contained in In_2_(S,O)_3_ films is not generated from single layers of In_2_O_3_ but rather by exchange reactions as described in the previous section. Indeed, the O_2_ plasma can directly react with the film surface and induce an exchange reaction with surface sulfur atoms. Figure 7 presents a scheme of the assumed substitution mechanism at the surface.

The following exchange reactions can explain the substitution of S atoms by reactive oxygen species generated in the plasma. Indeed, their free standard enthalpies all have negative values:

[2]
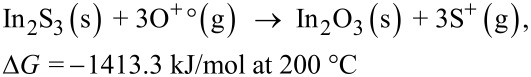


[3]
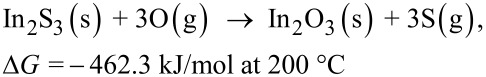


[4]
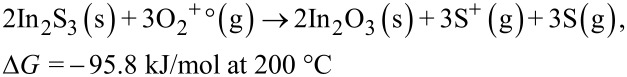


Thus, when comparing these reactions with the reaction between In_2_S_3_ and H_2_O, it seems that the doping is only favorable when using O_2_ plasma as oxygen precursor, because these reactions are all exothermic. This thermochemical analysis and the observation that In_2_(O,S)_3_ films obtained from the two different ALD pulse programs 20·{In_2_S_3_} + 2·{In_2_O_3_} and 20·{In_2_S_3_} + 2·O_2_ plasma have similar properties, show the critical role of activated oxygen during the deposition of In_2_(S,O)_3_.

Commonly existing species in oxygen plasmas are atomic oxygen that is created from molecular oxygen dissociation, excited oxygen species at different electronic levels, ionized oxygen or recombined species like O_3_ [32–33]. Consequently, exchange reactions between adsorbed oxygen and oxygen species from the gas phase or recombination reactions have to be considered. Marinov et al. studied the interactions between a radiofrequency O_2_ plasma and oxide surfaces like TiO_2_, SiO_2_ and Pyrex [32]. They demonstrated that these materials surfaces are continuously re-structured under O_2_ plasma exposure because of the exchange reactions that occur between O atoms in the films and oxygen species of the gas phase. They also reported that reaction products undergo oxidation at the surface and assumed that two surface mechanisms could occur; O + O → O_2_ and O + O_2_ → O_3_. Such mechanisms might also occur in our case considering the exchange reactions described in Figure 7 during the first O_2_ plasma pulse. Indeed, no match was found between the ratios of In_2_O_3_ cycles during the deposition of In_2_(S,O)_3_ films and the oxygen content of the films determined by EDX. When the number of In_2_O_3_ cycles varied from 4.8% to 11.8%, in the same time the oxygen content of the films varied from 68 atom % to 75 atom %. Oxidation mechanisms during deposition process can explain these high differences between the expected values and those measured.

Comparing In_2_(S,O)_3_ films synthesized with and without an In(acac)_3_ pulse before the O_2_ plasma pulse, no significant differences in the band gap values or the atomic ratios of the samples were observed. This confirms that the formation of an In_2_O_3_ single layer is not required to synthesize In_2_(S,O)_3_ films. These results are in fair agreement with the fact that the growth of pure In_2_O_3_ remained unsuccessful. Thus, we can assume that only activated oxygen is involved during the deposition of In_2_(S,O)_3_, and In_2_S_3_ can be considered as an intermediate state for the formation of In_2_(S,O)_3_.

On-going studies focus on a better understanding on the nature of the oxygen species generated by the plasma, their role in oxidizing mechanisms and the reason of the relatively low indium content. One of them could be an excessive adsorption of oxygen in the film and the formation of sulfates. Further studies, in particular by using X-ray photoelectron spectroscopy, are in progress to assess the presence or not of such groups. Experiments will also be performed to study the influence of other oxygen sources such as O_2_ alone and O_3_.

## Conclusion

In this study we reported the atomic layer deposition of In_2_(S,O)_3_ films by using In(acac)_3_ (acac = acetylacetonate), H_2_S, and either H_2_O or O_2_ plasma as oxygen sources. In_2_(S,O)_3_ films could only be obtained with O_2_ plasma as oxygen source, and all attempts to synthesize In_2_O_3_ remained unsuccessful. Thus, synthesis of In_2_(S,O)_3_ films is likely to occur through an exchange reaction instead of simple mixing of In_2_S_3_ and In_2_O_3_ layers. A thermochemical analysis can explain such observations. Indeed, this reaction is endothermic for H_2_O and exothermic for O_2_ plasma.

With this new synthesis method, the optical band gap of the thin films could be tuned from 2.2 eV to 3.3 eV by increasing the number of O_2_ plasma pulses. The high oxygen contents measured in the films (>66 atom %) in comparison to the initial number of In_2_O_3_ pulses might be explained by the fact that oxidation mechanisms occurred on the film surfaces during the O_2_ plasma pulses. Due to the reactivity of the plasma, the film surfaces cannot be considered as a static system but should rather be seen as continually re-structured surfaces. In our future studies, those films will be applied as buffer layer in Cu(In,Ga)Se_2_ solar cells to investigate their suitability as Cd-free buffer layer for thin film solar cells.

## Experimental

In_2_S_3_ and In_2_(S,O)_3_ thin films were deposited on borosilicate glass and Si(100) substrates in a SUNALE R-200 ALD reactor (Picosun Oy.) with a modified 15 cm × 15 cm square reaction chamber. All samples were deposited performing a total of 2000 growth cycles. The source material for indium was indium acetylacetonate (In(CH_3_COCHCOCH_3_)_3_), In(acac)_3_, (98%, Strem Chemicals). Hydrogen sulfide, H_2_S (99.5%, Messer) was used as the sulfur source. Deionized Millipore vapor water and O_2_, (99.9995%, Messer) were used as oxygen source. O_2_ was introduced in a remote RF plasma generator with Argon (99.9997%, Messer) as carrier gas, and the plasma power was kept at 2600 W. All sources were kept at room temperature while In(acac)_3_ was heated to 200 °C. The carrying and purge gas was nitrogen with a purity of 99.9999% (Messer). The pressure in the reaction chamber was kept in the range from 1 to 4 mbar.

The thickness of the films was measured using a VEECO DEKTAK 6M profilometer on glass substrates. Thicknesses were determined after creating steps in the films, by masking film parts with chemically resistant tape and dipping the film in nitric acid (45% in water) at room temperature for 60 s. The uncertainty given for the thickness is the standard deviation of six measurements taking into account the uncertainty of the profilometer, the sharpness of steps, the film roughness, and the film inhomogeneity. Transmittance and reflectance spectra were obtained by using a PerkinElmer lambda 900 Spectrophotometer with a PELA-1000 integrating sphere. All optical measurements were performed on borosilicate glass substrates. X-Ray diffraction (XRD) studies were performed under grazing incidence X-ray diffraction conditions with a PANalytical Empyrean diffractometer while using Cu Kα radiation. X-Ray reflectometry analyses were also performed to confirm thickness measurements. Thin film compositions were obtained by using a Magellan 400L scanning electron microscope provided by FEI. It is equipped with an energy dispersive X-ray spectroscopy detector INCASynergy 350. All EDX measurements were carried out on Si(100) substrates and the values reported are atomic percentages (atom %).
